# Supplementary material for: Experimental analysis of the stick-slip characteristics of faults at different loading rates
Source: PLoS One. 2020 Apr 24;15(4):e0231464. doi: 10.1371/journal.pone.0231464 (PMC7182388; doi:10.1371/journal.pone.0231464)
Supplement: Attachment — Submitted filename: comments for the tgeo-2019-0111 paper.docx [file pone.0231464.s001.docx]

Comments for the PONE-D-19-35929 paper “Experimental Analysis of Stick-Slip Characteristics of Faults at Different Loading Rates” by Jia et al.

This manuscript focuses on the fault stick-slip at different loading rates, and double-sided shear tests were conducted and the digital speckle correlation method were adopted for analysis. Besides the description of relevant experimental results, this article lacks theoretical analysis. Although the authors cited the empirical formulas of relevant scholars, they did not carry out an in-depth comparative analysis or not obtained new formulas containing the loading rate factor for reference. Therefore, this article also needs to be greatly improved and modified. Major revision is suggested. In addition, there are some details that need to be modified, as follows:

1)
Some citations cannot be found in the references. Please check the full text carefully, for example: Guo et al.(2014).

2)
More stick-slip stages are suggested to illustrate in Table 1 and Figure 3, only three stick-slip stages are not enough.

3)
Fig. 9 shows the relationship between the magnitude and the actual stress drop under different loading rates, but there are so little points for some loading cases, such as 8,10,15μm/s. In addition, these data show that linear fitting may not be appropriate, please consider it.

4)
Lines 221 to 227, please avoid this writing style, you can add a chart to explain it. Moreover, “For a given specimen, the slip displacement of the fault stick-slip increased as the stress drop increased.” In opinion of the reviewer, the stress drop should attribute to the sudden increase in displacement, not the opposite.
