# Supplementary material for: Experimental analysis of the stick-slip characteristics of faults at different loading rates
Source: PLoS One. 2020 Apr 24;15(4):e0231464. doi: 10.1371/journal.pone.0231464 (PMC7182388; doi:10.1371/journal.pone.0231464)
Supplement: Attachment — Submitted filename: Response to Reviewers.docx [file pone.0231464.s002.docx]

**Re:** Response to Reviewers

Manuscript Number: PONE-D-19-35929

Title: Artificial seismic source field research on the impact of the number and layout of stations on the microseism location error of mines

**Date:** December 5, 2018

Dear Editor,

Thank you for providing the reviews for our paper: No. PONE-D-19-35929 We would like to thank the reviewers for their time and consideration. Please find below (in red) direct responses to the reviewer comments. Thank you for the opportunity to revise the manuscript. We hope the changes listed have made the manuscript suitable for publication and we look forward to your response.

-Reviewer 1

The authors conduct an interesting experimental study on stick-slip characteristic of faults at different load rates, which is extensively and intensively used in underground mine operations worldwide. As reviewer, I am very pleased to suggest the manuscript to be accepted for publication through aforementioned minor revisions. All comments and suggestions are as follows;

1) The English of the paper needs some editing. There are many typos in the paper
 Authors’ response:

Thank you for your suggestion. It has been modified as required in the manuscript.

2) Introduction
The introduction is well articulated and the objective is well defined. However, the English needs to be edited

Authors’ response:

Thank you for your suggestion. It has been modified as required in the manuscript.

3) Materials and Methods
The English needs to be edited

Authors’ response:

Thank you for your suggestion. It has been modified as required in the manuscript.

4) Please note, too many details are missed in this section. Why you choose the granite rock as the materials? It also didn’t give the interface roughness information which is important for evaluating the slide characters. The load equipment details, and why you choose such a series of load rates and a 25MPa vertical load? Moreover, give more details about the CCD and high speed camera.

Authors’ response:

Thank you for your question.

(1)Granite is the most widely distributed in the stratum.The fault slip encountered in practical engineering generally occurs in the geological conditions of granite.It is universal to choose granite as experimental material.

(2)Under the condition of 25MPa vertical load, the roughness has little effect on the friction coefficient, and has great effect on the friction property. The main research object of this paper is the characteristics of fault stick slip. The main variable in the experiment is the loading rate. From the following calculation formulas,t can be known that the interface roughness is not a calculation parameter when calculating the magnitude of fault stick-slip and the stress drop.So it is not necessary to give the interface roughness.

(3)The load equipment details have been added in the Materials and Methods section.	The content is as follows:“The experimental loading device was a RLJW-2000 hydraulic servo testing machine with a maximum axial load of 3000 kN and a maximum horizontal load of 1500 kN. The data recording frequency range of the loading device was 1×10^−5^ to 30 Hz; and the strain loading rate was 1×10^−7^ to 1×10^−2^ mm/s.”

(4)The higher the loading rate, the more stable the sliding.The lower the loading rate, the more unstable the sliding, and the more prone to stick-slip. Therefore, when the loading rate is small, the speed rate of each test increases less.

(5)When the loading rate is greater than 10, the speed rate of each test increases greatly.When the vertical pressure is less than 20 MPa, the surface roughness of the sample has a greater effect on the friction coefficient. When the vertical pressure is greater than 30 MPa, the sample will have some deformation. 25 MPa selected by comprehensive consideration.

(6)Details of CCD cameras and high-speed cameras have been added in the materials and methods section.The content is as follows:“CCD is the most commonly used image sensor for machine recording. In this experiment, the model of the CCD camera used was acA1600-20gm.”and “The high-speed camera was an industrial digital camera, which converted digital image capture targets into image signals and sent them to a dedicated image processing system. The image system performed various operations on these signals to extract the features of the targets. The high-speed camera model used in this experiment was a Photron Fast Cam SA1.1. ”

6) Results and Discussion
The English needs to be edited, and you should explain what’s driving the phenomenon.

Authors’ response:

Thank you for your suggestion. It has been added as required in the Conclusions.The content is as follows:“(4) As the loading rate increased, although the sliding properties of the fault did not change, the magnitude and the stress drop of the fault also exhibited the characteristics of stable sliding.”

8) The results about the first stick-slip time varying the load rate are already well-known; they are not new. The reviewer suggests to the author to get more familiar with deformation and stress state in the interface which are responsible for the stick-slip character.

Authors’ response:

Thank you for your suggestion. It has been modified as required in the Conclusions section of the manuscript.

9) The results is lack of the mechanisms that can explain the phenomenon, the author should give more details.
 Authors’ response:

Thank you for your suggestion. Theoretical analysis and interpretation were added to the Results and discussion.The content is as follows:lines 208 to 218,lines 233 to 238,lines 270 to 275,lines 301 to 314.

-Reviewer 2

This manuscript focuses on the fault stick-slip at different loading rates, and double-sided shear tests were conducted and the digital speckle correlation method were adopted for analysis. Besides the description of relevant experimental results, this article lacks theoretical analysis. Although the authors cited the empirical formulas of relevant scholars, they did not carry out an in-depth comparative analysis or not obtained new formulas containing the loading rate factor for reference. Therefore, this article also needs to be greatly improved and modified. Major revision is suggested.

Authors’ response:

Thank you for your suggestion.Theoretical analysis and interpretation were added to the Results section of the manuscript.The content is as follows:lines 208 to 218,lines 233 to 238,lines 270 to 275,lines 301 to 314.

In addition, there are some details that need to be modified, as follows: Some citations cannot be found in the references. Please check the full text carefully, for example: Guo et al.(2014).
Authors’ response:

Thank you for your suggestion.We modified the format of the references.

2)More stick-slip stages are suggested to illustrate in Table 1 and Figure 3, only three stick-slip stages are not enough.
Authors’ response:

Thank you for your suggestion.We changed the original three stick-slip stages to six.

3)Fig. 9 shows the relationship between the magnitude and the actual stress drop under different loading rates, but there are so little points for some loading cases, such as 8,10,15μm/s. In addition, these data show that linear fitting may not be appropriate, please consider it.
Authors’ response:

Thank you for your suggestion.As the loading rate increases, the sliding will tend to be stable, and the number of stick-slips will decrease. So there are fewer points. The fitted lines for the points of 8, 10, and 15 μm / s are yellow, green, and red line segments, as shown in the following figure. We can find that the two fit well.

4)Lines 221 to 227, please avoid this writing style, you can add a chart to explain it. Moreover, “For a given specimen, the slip displacement of the fault stick-slip increased as the stress drop increased.” In opinion of the reviewer, the stress drop should attribute to the sudden increase in displacement, not the opposite.

Authors’ response:

Thank you for your suggestion.We changed “For a given specimen, the slip displacement of the fault stick-slip increased as the stress drop increased.” to “For a given specimen, the increase in the stress drop occurred due to the sudden increase in the slip-slip displacement of the fault.”

In addition, we received some suggestions from the editor.

1.Please amend your Methods section to include the origin and vendor information and other characteristics of all materials and equipment used, in enough detail for another researcher to replicate the findings.

Authors’ response:

Thank you for your suggestion. It has been modified as required in the materials and methods section of the manuscript.

2.PLOS requires an ORCID iD for the corresponding author in Editorial Manager on papers submitted after December 6th, 2016.

Authors’ response:

Thank you for your suggestion. We have added ORCID ID in the Editorial Manager .

We have marked the changes in the uploaded 'Revised Manuscript with Track Changes' as red.

Please feel free to contact me with any questions or considerations.

Sincerely,

Baoxin Jia
